# Considerations of Racism and Data Equity Among Asian Americans, Native Hawaiians, And Pacific Islanders in the Context of COVID-19

**DOI:** 10.1007/s40471-022-00283-y

**Published:** 2022-03-18

**Authors:** Gilbert C. Gee, Brittany N. Morey, Adrian M. Bacong, Tran T. Doan, Corina S. Penaia

**Affiliations:** 1 Department of Community Health Sciences, University of California Los Angeles, 650 Charles E. Young Drive South, Los Angeles, CA USA; 2 Department of Health, Society, & Behavior, Program in Public Health, University of California Irvine, 653 E. Peltason Dr., Anteater Instruction and Research Building (AIRB) 2022, Irvine, CA USA; 3 Department of Health Management and Policy, University of Michigan, 1415 Washington Heights, Ann Arbor, MI USA; 4 Asian Pacific Islander Forward Movement, 905 East 8th Street, Los Angeles, CA USA; 5 Department of Health Policy and Management, University of California Los Angeles, 650 Charles E. Young Drive South, Los Angeles, CA USA

**Keywords:** Asian Americans, Native Hawaiians, Pacific Islanders, COVID-19, Racism, Discrimination, Disparity

## Abstract

**Purpose of Review:**

The COVID-19 pandemic has revealed the importance of considering social determinants of health, including factors such as structural racism. This review discusses some of the evidence that triangulates on this issue, including data from hate crime statistics, social media analysis, and survey-based research. It also examines the data needs for Asian Americans, Native Hawaiian, and Pacific Islander (NHPI) communities.

**Recent Findings:**

The available data provides evidence that the pandemic has contributed to an increase in anti-Asian sentiment and discriminatory incidents. Many reports have surfaced showing a surge in anti-Chinese discrimination, which has “spilled over” into other Asian communities. Research is beginning to emerge to show that such discrimination may also impact health issues such as psychological distress. Given prior research, we would expect many more studies to emerge in the future. Also, the pandemic has illustrated the major gaps in data available to disentangle the health and social concerns facing Asian Americans and NHPI communities. Significant issues include the lack of systematic reporting of data for these communities both across states, and even among agencies within a state; erroneous aggregation of Asians with NHPIs; and censoring of data. These gaps and issues contribute to bias that obscures objective data and amplifies health inequalities.

**Summary:**

The COVID-19 pandemic has had a negative impact on the well-being of Asian American and NHPI communities. It is critical to provide disaggregated data, not only so that we can have accurate reporting, but also to ensure data and health equity.

## Introduction

Although social and medical issues are often siloed into discrete professions, the COVID-19 disease has highlighted their tight interdependence. This disease has led to ripple effects on politics, education, employment, housing, and all other facets of life. One important consideration is race relations and racism [1, 2]. In this paper, we use COVID-19 to illustrate 3 key themes related to racism and health of Asian American, Native Hawaiian, and Pacific Islander (NHPI) communities: (1) racism is a major public health issue; (2) racism is not simply about interpersonal experiences of hate crimes or micro-aggressions; (3) disaggregation is paramount. Due to the evolving nature of the pandemic, our review encompasses not only traditionally peer-reviewed articles, but also working papers, newspaper reports, and other non-peer-reviewed resources.

## Racism and Health

Racism is a major public health issue that continues to affect Asian and NHPI communities in the United States (U.S.) [3, 4]. Racism can be defined as the totality of how social systems, ideologies, and practices are organized so as to maintain white supremacy [5, 6]. The COVID-19 pandemic has shown this effect clearly. Since the development of the pandemic in 2020, several different types of data show an increase in anti-Asian bias [7].

According to the California Health Interview Survey, in May 2020, about 5.4% of Asians in the state reported unfair treatment due to COVID-19 compared to only 0.4% of Whites. Considering there are about 4.9 million Asian Californians, this could translate to a quarter-million Asians who experienced discrimination due to COVID-19 in that state alone [8]. Furthermore, these estimates may be underestimated. A study of Chinese American families found that half of parents and children experienced such discrimination [9].

A study by Nguyen et al. analyzed negative racial sentiment appearing in 2.3 million tweets from November 2019 to April 2020 [10]. Negative sentiment was stable across this period for all racial groups except for Asians, which rose by 55% in just 1 month, from February to March (by comparison, it rose by 5% for African Americans and declined by 4% for LatinX). March 2020 was particularly important because that was when the U.S. began to seriously grapple with the pandemic. It also represented the beginning of politicians using harmful rhetoric that has fueled Asian-based racism. On March 7, 2020, former U.S. Secretary of State Mark Pompeo used “Chinese virus” and on March 8, Congressman Paul Gosar used “Wuhan Virus” in public appearances. One report found an 800% growth in the use of these terms on online news outlets by March 8 [11]. Darling-Hammond and colleagues corroborated this when they investigated implicit (unconscious) bias against Asians using national data from Project Implicit [12]. They found that implicit bias against Asians has been declining since 2007, but began rising after March 8. Another study that polled 1141 adults in the U.S. indicated that 42% of respondents were “somewhat or very likely” to discriminate against Asians because of COVID-19 [13]. Such discrimination included things such as sitting next to an Asian person on a bus or being at a restaurant with primarily Asian staff. Thus, the rise of derogatory language in tweets and political rhetoric may contribute to normalizing biases against Asians in society.

On March 16, 2020, the discussions amplified when former President Trump tweeted: “The United States will be powerfully, supporting those industries, like Airlines and others, that are particularly affected by the Chinese Virus. We will be stronger than ever before!” The use of phrases such as “Chinese virus” evokes considerable controversy due to the stigma it elicits towards a particular people group and country. However, the president and others defended the use of “Chinese virus,” saying that it was simply a descriptor of the pathogen’s geographic origins.

Research by Hswen et al. investigated the extent to which the hashtag #Chinesevirus was associated with more hate speech compared to #COVID-19 [14]. The analyses focused on the week prior to Trump’s tweet versus the week after. The data indicated two key findings: (1) over 50% of the hashtags associated with #Chinesevirus were associated with hate speech, compared with only 20% of those with #COVID-19; (2) #COVID-19 was the more commonly used hashtag prior to Trump’s tweet, but #Chinesevirus became more prevalent after; (3) the number of anti-Asian hashtags grew, from about 12,000 before Trump’s tweet, to over 400,000 after.

On March 23, 2020, the former president said, “They’re amazing people and the spreading of the virus is not their fault in any way, shape or form.” Afterwards, the former president continued to use phrases such as “kung flu” that have been considered prejudicial and offensive by many Asian Americans [15]. It would be unreasonable, however, to attribute all of the anti-Asian sentiment to the president. Many other influential leaders in the U.S. and around the world have issued prejudicial remarks, including: “China is to blame because the culture where people eat bats & snakes & dogs & things like that,” by Senator John Cornyn. Similar reports were raised in other countries, including the UK, Australia, and Canada [16].

**Fig. 1:**
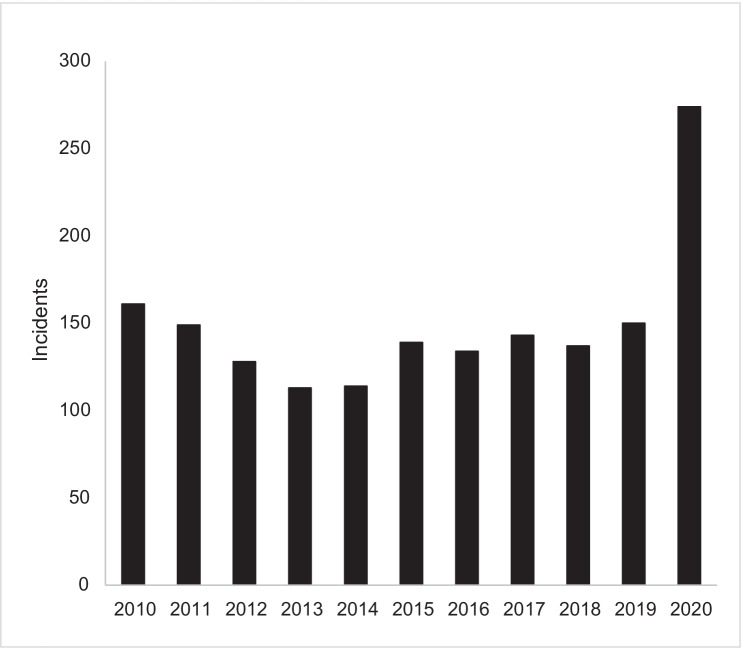
Anti-Asian Hate Crimes reported by the FBI, 2010–2020 (source: Federal Bureau of Investigation Crime Data Explorer. Data downloaded 10/22/21)

There has been a marked rise in Asian victims of hate crimes in the U.S. On March 27, 2020, the Federal Bureau of Investigations issued a warning to law enforcement agencies about a surge in anti-Asian hate crimes [17]. Figure 1 shows a count of anti-Asian hate crimes from 2010 to 2020. The reporting of hate crimes is relatively constant between 2010 and 2019 (mean = 137), but doubles to 274 in 2020. We caution, however, that FBI hate crimes are likely underreported due to various factors including law enforcement officials not classifying some incidents as hate crimes, language barriers, and distrust of some community members against law enforcement entities. A key example was the Atlanta spa shootings, where 6 of the 8 victims killed were Asian women. These murders were not classified as a hate crime. Indeed, at a press conference, the sheriff’s department characterized the shooter as “he was pretty much fed up… and I guess it was a really bad day for him, and this is what he did.” [18] Comments such as these, which appear to show more sympathy for the shooter than for the victims, contribute to community distrust of formal channels.

In response to the rise in anti-Asian incidents, members of the API community created the stopaapihate.org website in March of 2020 to provide a more accessible and credible reporting resource. They have collected 9018 incidences from March 19, 2020, to June 30, 2021. They note that their largest increase in reports occurred from April to June 2021, indicating the persistent ongoing occurrence of hate crimes as the pandemic continues.

Significantly, although many of these incidents are purportedly anti-Chinese, there have been spillover effects against other Asian groups both in the U.S. and abroad. For example, Japanese and Vietnamese people in Finland have been targets of anti-Asian hate incidents related to COVID-19 [19]. StopAAPIHate.org reported that Chinese-identifying people made up the largest proportion of hate report incidents (43.5%) followed by Koreans (16.8%), Filipinx (9.1%), Japanese (8.6%), and Vietnamese (8.2%) [20]. This conflation of Asian ethnicities is likely due to the “they all look alike” phenomenon [21].

Thus, it seems reasonable to conclude that anti-Asian bias did increase as a result of the pandemic and that statements using stigmatizing language such as “Chinese virus” contributed to it. This conclusion is based on triangulating evidence from various sources, including the rise in implicit biases, and the growth of derogatory tweets and hashtags, and increase in hate crimes.

In a world that is interconnected through social media and Internet media outlets, the rapid spread of anti-Asian racism is a global phenomenon [22]. Past research has largely focused on within-country racism, such as how discrimination affects health among aboriginal populations in Australia or Black persons in South Africa [23–25]. However, the literature could be expanded to consider how racism operates worldwide by examining globally connected racist organizations and systems. This would allow the literature on racism and health to join other pieces of literature, such as that regarding colonization and Empire [26, 27].

## Current Tropes Reflect Historical Processes

The discrimination uncovered by COVID-19 is not a new phenomenon. Rather, it continues the “yellow peril” tropes established centuries ago [28]. The SARS-Cov2 virus was originated in China in December 2019. In January 2020, newspapers began reporting on anti-Asian hate incidents in the U.S. and other countries. By June 2020, reports showed that many of the U.S. COVID-19 cases originated in Europe [29]. This information did not appear to reverse discrimination against Asians nor increase discrimination against Europeans.

Why would any disease be linked to an ethnic group? The simple answer is because we have a history of doing so. Racial groups have long been associated with diseases. This includes so-called illnesses such as “Draptomania,” referring to the illness of slaves running away from their masters to the relatively newer condition of “Chinese restaurant syndrome,” referring to the headaches and other problems associated with eating Chinese food [30, 31]. Both of these conditions have been debunked, but nonetheless, the theme of racial groups being the originators of specific illnesses remains prevalent.

Currently, we are facing a similar situation with the association of COVID-19 as the “Chinese virus” In modern history, we have witnessed similar labels, such as the term, “Gay-Related Immune Deficiency” (GRID) (subsequently relabeled as “Acquired Immunodeficiency Syndrome; AIDS”) and Middle-East Respiratory Syndrome (MERS) [32]. In 2015, recognizing that such labels led to stigma and social harm, the World Health Organization (WHO) issued guidance that new diseases should not be named for specific communities, geographic origins, and similar factors [33]. The WHO has stated that neutral scientific phrases should be used in lieu of terms such as “Chinese virus,” to avoid stigmatizing people or places.

A key problem is that in public health and medicine, we have a dual view of what race signifies [34–36]. On the one hand, many have espoused that race is a “social construct,” reflecting membership in socially defined groups rather than intrinsic biological characteristics. On the other hand, many of our practices reflect a tacit assumption that race is a biological characteristic. For example, the FDA approval of the drug Bidil for African Americans only, but for no other races, provides implied agreement that race is biology [37].

Because the scientific community cannot provide a consistent understanding of race, it is not surprising that the public also holds multiple viewpoints. Many of these views reflect the vestigial notions from centuries ago that have never left our consciousness that link race with biology and biology with disease [28]. Given these proclivities, it is conceptually “easy” and socially acceptable to blame Asians for the current pandemic.

## Structural Racism Manifests in Very Subtle, But Consequential Ways

COVID-19 illustrates how quickly and easily we can spread prejudicial ideas, which are likely amplified by political entities. Furthermore, the pandemic reminds us about the historical roots of these racist ideas. The durable and widespread nature of these effects cannot be due to prejudices of individuals alone, but rather, reflects the deeper problem of structural racism [4, 38]. The literature has shown that structural racism affects the key social determinants of health, including things such as residential location, educational trajectories, accumulation of wealth, gainful employment, access to medical care, and so forth [38, 39].

However, discrimination manifests in very mundane processes that can be quite consequential. We illustrate this idea with regard to the collection, reporting, and coding of racial data. Consider Table 1, which illustrates how race is computed for infant births and deaths in Texas [39]. Although data for Asians and NHPIs are collected, they are ultimately classified as “other/unknown” along with American Indians, Alaskan Natives, multiple races, and missing. Because this “other” category is so heterogeneous, it is often eliminated from reports. Thus, Asians and NHPIs are rendered invisible.

**Table 1:** Race/ethnicity computation for Texas birth and death events

If race is reported as:	And Hispanic Origin is reported as:	Then race/ethnicity is computed as
White	Non-Hispanic, not classifiable	White
Black	Non-Hispanic, not classifiable	Black
Any single race or multiple races	Mexican, Puerto Rican, Cuban, other	Hispanic
Asian, American Indian or Alaskan Native, Native Hawaiian or other Pacific Islander, other, multiple races, blank, or unknown	Non-Hispanic, not classifiable	Other/unknown

Source: https://www.dshs.texas.gov/chs/vstat/vs09/t44.shtm

Accessed: 9/1/20

As Table 2 shows, only 40 states provide any information about Asians and only 23 provide information about NHPIs. Although a common reason for omitting Asians and NHPIs in reports is due to concerns about small samples and desires to protect the confidentiality, other reasons should be considered. Indeed, COVID-19 illustrates that this is much an issue of political will as it is anything else.

**Table 2:** Reporting of Asian American, Native Hawaiian, and Pacific Islander data for COVID-19 infections as of 9/13/20 by State (Inclusive of Washington, District of Columbia and the U.S. Virgin Islands)

State	Reports data on Asians Separately	Reports data on Native Hawaiians and Pacific Islanders separately	Aggregates Asians, Native Hawaiians, Pacific Islanders as one category	Aggregates Asians or Native Hawaiians and Pacific Islanders in “other” category
Alabama	X			X
Alaska	X	X		
Arizona			X	
Arkansas	X	X		
California	X	X		
Colorado	X	X		
Connecticut			X	
Delaware			X	
D.C.	X	X		
Florida				X
Georgia	X	X		
Hawaii	X	X		
Idaho	X	X		
Illinois	X	X		
Indiana	X			X
Iowa	X	X		
Kansas	X			X
Kentucky	X	X		
Louisiana	X	X		
Maine				
Maryland	X			X
Massachusetts	X			X
Michigan			X	
Minnesota	X	X		
Mississippi	X			
Missouri				X
Montana	X			
Nebraska	X	X		
Nevada	X			X
New Hampshire	X			X
New Jersey	X			X
New Mexico	X			
New York			X	
North Carolina	X	X		
North Dakota	X			X
Ohio	X	X		
Oklahoma			X	
Oregon	X	X		
Pennsylvania	X			X
Puerto Rico				
Rhode Island	X			X
South Carolina	X			X
South Dakota	X			X
Tennessee	X	X		
Texas	X			
Utah	X	X		
Vermont	X			X
Virgin Islands				
Virginia			X	
Washington	X	X		
Wisconsin			X	
West Virginia		X		X
Wyoming	X	X		
**Count**	**40**	**23**	**8**	**17**

Note. Data were taken from COVID Racial Data Tracker which records COVID-19 case and mortality data for all 50 U.S. States, the District of Columbia, and associated territories. Numbers reflect available data up to September 23, 2020. Data are publicly available and gathered through state health department websites

For example, on April 29, 2020, only 27 states reported COVID-19 infections data on Asians, 15 reported data on NHPIs, and 8 states reported Asians and NHPIs aggregated together [40]. However, a mandate was passed in June 2020 that required states to disaggregate [41]. By September 13, 2020, 40 and 23 states reported separate data on Asians and NHPIs, respectively (and 8 reported Asians and NHPIs aggregated together). Similar findings are seen for mortality data: from 26 to 43 states for Asians and 9 to 15 states for NHPIs [40].

The lack of systematic reporting can be seen in the examples of Pennsylvania and Oregon. These states have similar proportions of Asian Americans (3.4% and 4.9%, respectively in 2019) and NHPIs (0.1% and 0.5%, respectively); however, their State Health Departments used different categories for reporting race. In Pennsylvania, vital statistics were reported for White, Black, Hispanic Origin, Asian/Pacific Islander, and multi-race [42]. In contrast, Oregon reported vital statistics for White, Black, Hispanic, American Indian, Asian, NHPI, other, and, not stated, multiple races [43]. Pennsylvania combined Asian and NHPIs and does not discuss which specific subgroups comprise this category. However, Oregon clarified Asians to comprise Indian, Chinese, Filipino, Japanese, Korean, Vietnamese, and other Asian. Oregon also indicated that NHPIs include Guamanian, Hawaiian, Samoan, and other Pacific Islanders.

Unfortunately, Oregon—along with California, Hawaii, Utah, and Washington—is the exception in publishing vital statistics for Asians and NHPIs separately. Considering that Asians and NHPIs are often lumped together in the majority of states’ vital statistics reporting and knowing that COVID-19 disproportionately impacts NHPIs, most states’ Department of Health do not have data reporting infrastructures that provide usable estimates of vital statistics (such as COVID-19-related deaths) within Asian and NHPI subgroups. Additionally, over half of NHPIs are multiracial, but identify as NHPIs, causing this population to be severely undercounted.

Furthermore, within states, the various reporting agencies do not report race/ethnicity data consistently. For example, Florida’s Department of Education provides data on White, Black or African American, Hispanic/Latino, Asian, American Indian or Alaska Native, two or more races, and Native Hawaiian or other Pacific Islander. However, the Florida Department of Health’s vital statistics reports resident live births as White, Black, or unknown/other [44]. Thus, we do not have coherent information within the same state to comprehensively assess health and the social determinants of health [45].

Individual researchers also make decisions that can have a disparate impact on Asian populations. For example, one article used a “hierarchical approach to assign a single-mutually exclusive” race/ethnicity to research participants [46]. This approach first assigned all participants to Black if that race was mentioned. Thus, if a participant was Asian and Black, they would be classified as Black. Next on the hierarchy was Hispanic, which took all mentions of Hispanic (except Black). Third were Asians, and finally Whites. This decision was consequential, as the authors noted, “Compared to the general population in the Bay Area, our sample … had greater representation of Hispanics and Blacks, and fewer Asians.” Plausibly, had they used a different decision rule, their sample may have been more representative. What is important is that the statistical hierarchy likely reflected an unstated racial hierarchy. As far as race matters go, Asians and NHPIs are often left as an afterthought or assumed to represent a “model minority” that have overcome social inequalities [47].

This is where understanding the heterogeneity of the population is critical. Not only has the COVID-19 pandemic revealed how a society can use a disease as a weapon to discriminate against a group of people, but also, it has created an environment in which existing issues of structural racism have exacerbated the disease impact [48–50]. This is exemplified in the disproportionate burden of COVID-19 deaths among NHPIs [51, 52].

Although NHPIs are considered a separate race category from Asians by the Office of Management and Budget (OMB), NHPIs are often erroneously aggregated with Asians [8, 53, 54]. The OMB defines NHPI as a person having origins in any of the original peoples of Hawaii, Guam, Samoa, or other Pacific Islands [55]. Disaggregated data reveals that in the U.S., NHPIs are 2.9 times more likely to have died from COVID-19 than white Americans, accounting for age [40]. On September 15, 2020, the NHPI mortality rate of 72 per 100,000 is higher than that of Asians (40 per 100,000), non-Hispanic whites (47 per 100,000), and Latinxs (65 per 100,000), and is only below the rates for Native Americans (82 per 100,000) and Black Americans (98 per 100,000). Using the Asian and Pacific Islander or API “catch-all” hides these important NHPI health disparities in states and localities. Therefore, NHPIs should be consistently disaggregated from Asians in disease reporting [8].

The reporting of Asians as a monolithic group also hides disparities among the diverse sub-populations [45, 56, 57]. Disaggregated data in California reveals that over 40% of COVID-19 deaths among the state’s Asian Americans are Filipino, although they make up only 20% of the state’s Asian American population The high burden of death among Filipinos may be attributed to preexisting health conditions (e.g., diabetes, hypertension), living in nursing homes or multigenerational homes, and working in essential jobs, especially in healthcare. Nursing is a common occupation among Filipino Americans, which can be traced back to the colonial history of the U.S. in the Philippines [58, 59]. Filipinos have long been recruited to fulfill nursing shortages in the U.S. [60, 61]. Currently, this is putting many working-age Filipino Americans at risk for contracting and dying of COVID-19. These disparities among Asian American sub-populations are obscured by the lack of disaggregated data. More disparities likely exist.

Preliminary research reveals that in California counties where the highest percentage of Asian Americans are Southeast Asians (i.e., Burmese, Cambodian, Hmong, Indonesian, Laotian, Malaysian, Thai, and Vietnamese), the ratio of COVID-19 related deaths over total cases is highest among Asian Americans [52]. By comparison, if the percentage of East Asian (i.e., Chinese, Japanese, Korean, or Taiwanese) or South Asian (i.e., Asian Indian, Bangladeshi, Burmese, Nepalese, Pakistani, and Sri Lankan) among Asian Americans was high in counties, the ratio was lower.

Disaggregating NHPI people by subgroup is also important. Disaggregated data of the 2020 calendar year in California revealed that Samoans had the highest COVID-19 crude mortality rate among NHPI people (182 per 100,000), followed by Tongans (142 per 100,000) then Chamorros (107 per 100,000). The overall rate among NHPI was 123 per 100,000 while the aggregated rate for Asians and NHPI was 75 per 100,000. Thus, even reports of COVID-19 mortality by race group can hide subgroup disparities [62].

Aggregating data into the monolithic Asian or NHPI category hides inequalities, harms members of those communities, and stymies public health action to address disparities [8, 63]. Furthermore, disaggregating data is often a matter of political will [62]. Therefore, data aggregation and hidden data equates to another form of structural racism [64].

## Closing

The pandemic has reminded us that racial discrimination is inexplicably connected to health. The disparities literature has generally emphasized that discrimination contributes to stress, maladaptive coping behaviors, and illness [65–69]. Although research on COVID-19 related discrimination and health consequences is still emerging, this would be a predictable pattern given existing research [68]. However, the pandemic has also highlighted reverse causality—although we readily recognize that illnesses can lead to medical stigma (e.g., for problems such as mental illness), we rarely conceive that illnesses can lead to racial stigma. COVID-19 provides a clear illustration of this phenomenon.

Additionally, the pandemic further highlights the key differences between Asians and NHPIs and the variation within these diverse communities. Asians and NHPIs were traditionally considered together, and indeed, many researchers and organizations still combine both groups. However, NHPIs have rightly raised concerns that their communities are often overlooked when aggregated with Asians [8, 70, 71]. The stark differences in the rates of infection and death highlight these key differences. One important way to strengthen health equity is to consistently provide data disaggregated by race and subgroup in a meaningful manner and to work in collaboration with the communities being affected [52, 72–74].

Finally, our thoughts should not simply be about the current situation. COVID-19 has merely amplified the biases and inefficiencies that were already in existence [75, 76]. To avoid a similar situation in future situations, whether they be infectious outbreaks, natural disasters, or otherwise, we need to simultaneously attend to all of the system-related factors that contribute to the health of our populations and specifically to health inequities.
